# Ten-year trends and influencing factors of hospitalization costs in chronic obstructive pulmonary disease: a quantile regression analysis (2016–2025)

**DOI:** 10.3389/frhs.2026.1811807

**Published:** 2026-06-02

**Authors:** Jieyun Zhu, Min Gao, Xiaoning Meng, Qiuyun Song, Zihuan Peng, Qiaoyan Wang, Yi Han, Zhaoqiang Cai, Changguang Ye, Zhao Lu

**Affiliations:** 1 International Medical Department, The People’s Hospital of Guangxi Zhuang Autonomous Region, Guangxi Academy of Medical Sciences, Nanning, China; 2 Department of Respiratory and Critical Care Medicine, Hepu County People’s Hospital, Hepu, China

**Keywords:** chronic obstructive pulmonary disease (COPD), hospitalization costs, influencing factors, quantile regression, temporal trends

## Abstract

**Background:**

To investigate the composition, temporal trends, and factors associated with per-admission hospitalization costs for chronic obstructive pulmonary disease (COPD)—related admissions from 2016 to 2025, adjusted for inflation and temporal effects, and to inform clinical cost control and optimized management strategies.

**Methods:**

We analyzed 4,766 COPD admissions events to a tertiary hospital in Guangxi, China. Descriptive analysis characterized cost trends and composition, followed by nonparametric tests, multiple linear regression, and quantile regression to identify cost-influencing factors.

**Results:**

Among the 4,766 eligible admissions, 75.9% (*n* = 3,617) occurred in male patients, with a mean age at admission of 74.50 ± 9.92 years. The overall comorbidity prevalence per admission was 97.80%, showing a steady increasing annual trend. Inflation-adjusted total per-admission costs remained stable over the decade (median: 6,500.53 RMB), with drug, medical service, and diagnostic costs as the largest components. Univariate analysis showed that age, length of stay (LOS), admission route and status, discharge mode, comorbidity count, antibiotic use, surgery/invasive procedures, admission year, and specific comorbidities including hypertension, heart disease, cerebral stroke, asthma, chronic lung disease, and kidney disease were significantly associated with per-admission hospitalization costs (all *P* < 0.05). Multiple linear regression identified significant admission year effects (2017, 2022, 2023, and 2024 as positive predictors, and 2021 and 2025 as negative predictors), age, LOS, comorbidity count, emergency admission, critical illness, invasive procedures, and cardiac/cerebral comorbidities as significant independent predictors (adjusted *R*^2^ = 0.703). Critically, quantile regression revealed that LOS, comorbidity count, invasive procedures, and emergency admission were associated with a more pronounced increase in costs among high-cost admissions, with regression coefficients increasing across quantiles.

**Conclusion:**

Inflation-adjusted per-admission COPD hospitalization costs were stable over the decade, driven by drugs, medical services and diagnostics. Admission year, age, comorbidity count, LOS, invasive procedures and acute critical admission correlated with higher costs, with stronger associations in high-cost admissions. These findings support the development of targeted interventions for cost containment and standardized clinical management of COPD.

## 1 Background

Chronic obstructive pulmonary disease (COPD) is a chronic airway disorder primarily defined by persistent airflow limitation (1). Its clinical course is chronic and progressive, and hospitalization for recurrent acute exacerbations not only inflicts severe physical and psychological distress on patients and increases the workload of healthcare providers, but also imposes a heavy burden on societal medical resources and the overall economy (2, 3).

In China, amid rapid population aging and persistent risk factors such as air pollution, the prevalence of COPD in adults aged 40 and over stands at 13.7% (3). Hospitalization is a key intervention for acute exacerbations of COPD, yet the 10-year dynamic changes in its hospitalization cost structure remain understudied. Most prior research on COPD-related medical costs in China has focused on short-term cross-sectional analyses of cost characteristics (4–6); decade-long longitudinal studies of these costs are scarce, with few targeted investigations in high-prevalence regions. As a high-prevalence COPD province in China, Guangxi has typical and representative regional medical cost patterns, and research here can provide valuable evidence for national COPD cost management strategies.

Besides, traditional analyses of medical cost determinants have mainly relied on ordinary least squares (OLS), generalized linear models (GLM), and multiple linear regression (7–9), which only capture average effects, lack outlier robustness for skewed cost data, and cannot reveal heterogeneous effects across cost tiers. In contrast, quantile regression models (10, 11) can accurately identify key factors at different cost levels. Leveraging this well-established health economics method, we applied it to a 10-year COPD dataset from a high-prevalence Chinese region, providing robust evidence for stratified, targeted cost-control policies.

Accordingly, to address the limitations of single-model analyses, we integrated multiple linear regression and quantile regression to comprehensively analyze the factors influencing per-admission hospitalization costs for COPD-related admissions. Using 10 years of consecutive data from a tertiary general hospital in Guangxi, China, our study not only delineated long-term trends in hospitalization costs but also clarified the heterogeneous impacts of these factors across different cost tiers. These findings identify key determinants of hospitalization costs, providing empirical evidence to inform cost-containment strategies and reduce the economic burden associated with COPD hospitalizations.

## 2 Materials and methods

### 2.1 Data source and study population

We retrieved and analyzed data from all COPD-related hospitalization events at a tertiary general hospital in Guangxi, China, from 2016 to 2025. The primary analysis unit of this study was individual hospitalization events, rather than unique patients, to accurately reflect real-world trends in per-admission costs and their influencing factors.

Inclusion criteria: Hospitalization events with a primary discharge diagnosis of COPD (ICD-10 codes: J44.0 and J44.1). Exclusion criteria: ① Incomplete key information (e.g., missing age, gender, or other baseline characteristics) or logical inconsistencies in medical records (e.g., discrepancies between the sum of individual cost components and total hospitalization costs); ② Length of hospital stay <24 h.

This study was approved by the Ethics Committee of Hepu County People's Hospital (Approval No.: 202303). All patient data were de-identified, and the study met the criteria for ethical exemption.

### 2.2 Outcome and covariates

Detailed data on all components of hospitalization costs were collected and categorized into six categories: comprehensive medical service costs, diagnostic costs, treatment costs, drug costs, consumable costs, and other costs. To ensure the reliability of cost analyses, all hospitalization costs (2016–2025) were adjusted for inflation using the Consumer Price Index (CPI) of Guangxi Zhuang Autonomous Region, with 2016 as the benchmark year to compute real constant prices. CPI data were retrieved from the National Bureau of Statistics of China.

All hospitalization costs in this study are reported in Chinese yuan (CNY, ¥). For international readers' reference, the average exchange rate of CNY to USD during the study period (2016–2025) was approximately 1 CNY = 0.14 USD (or 1 CNY ≈ 0.13 EUR, 2016–2025 average).

To identify factors associated with hospitalization costs, the following variables were included as explanatory variables:

Demographic factors: age, gender, marital status;

Disease-and hospitalization-related factors: primary and secondary diagnoses, number of comorbidities, admission year, admission status, admission route, number of prior hospitalizations, length of stay (LOS), discharge mode, history of surgery/invasive procedures, and antibiotic use.

Comorbidity was defined based on the presence of the following 14 common chronic diseases, adapted from the CHARLS database (12) (chronic lung disease excludes COPD, the study's primary diagnosis): hypertension; chronic lung diseases; diabetes mellitus or elevated blood glucose (including impaired glucose tolerance and elevated fasting blood glucose); malignant tumors (e.g., cancer, excluding mild skin cancer); heart diseases (e.g., myocardial infarction, coronary artery disease, angina pectoris, congestive heart failure, and other cardiac disorders); cerebral stroke (including hemorrhagic and ischemic subtypes); emotional and psychological disorders; arthritis or rheumatism; dyslipidemia (hyperlipidemia or hypolipidemia); liver diseases (excluding fatty liver, tumors, or cancers); kidney diseases (excluding tumors or cancers); gastric or digestive system diseases (excluding tumors or cancers); asthma; and memory-related diseases (e.g., senile dementia, brain atrophy, Parkinson's disease).

To ensure statistical reliability, total comorbidity count and prevalence were calculated across all 14 conditions for descriptive analysis. For inferential analyses (including univariate testing, multivariable regression, and quantile regression), we excluded conditions with prevalence <2% (arthritis or rheumatism, dyslipidemia, liver diseases, emotional and psychological disorders, and memory-related diseases) to mitigate bias from sparse data.

### 2.3 Statistical analysis

Categorical data were presented as frequencies (percentages), and group comparisons were conducted using the chi-square test or Fisher's exact test, as appropriate. Continuous data were reported as median (P25, P75) due to the skewed distribution, and comparisons were performed using the Mann–Whitney *U*-test for two groups or the Kruskal–Wallis *H*-test for three or more groups.

Given the skewed distribution of per-admission hospitalization costs, natural logarithmic (ln) transformation was performed for a pre-specified full multiple linear regression model (including all variables significant in univariate analysis) to meet normality assumptions. Normality of the ln-transformed hospitalization costs was then assessed via skewness and kurtosis statistics (|skewness| < 3, |kurtosis| < 10), confirming approximate normality for linear regression. Exponentiated coefficients [EXP(β)] are presented as geometric mean ratios (GMRs) to quantify relative cost changes. Quantile regression, robust to skewed data, was conducted on untransformed costs to explore heterogeneous effects at the 10th, 50th, and 90th percentiles.

All statistical analyses were conducted using R software (version 4.5.0). Two-sided statistical tests with *P* < 0.05 were considered statistically significant.

## 3 Results

### 3.1 Characteristics of the study cohort

A total of 4,766 eligible COPD-related hospitalization events were included, 75.9% of which occurred in male patients. The mean age at admission was 74.50 ± 9.92 years (range: 37–104 years), and 92.9% of admissions were for patients aged ≥60 years. The median number of hospitalizations per admission was 2 (mean: 3.98 ± 5.62, range: 1–57), showing a steady upward trend over the decade: from 1.33 admissions per patient in 2016 to 7.16 in 2025, with a notable acceleration after 2024. The median LOS was 9 days (mean: 10.61 ± 8.33, range: 1–89 days), following a “fluctuation–decline–recovery” pattern: it stabilized at ∼10 days (2016–2020), dropped to 8.42 days in 2021, then rose to a peak of 12.83 days in 2024, and slightly declined to 12.02 days in 2025. This fluctuation pattern aligns with the timeline of China's COVID-19 public health policies (2020–2022 dynamic zero-COVID period and subsequent relaxation).

**Figure 1:**
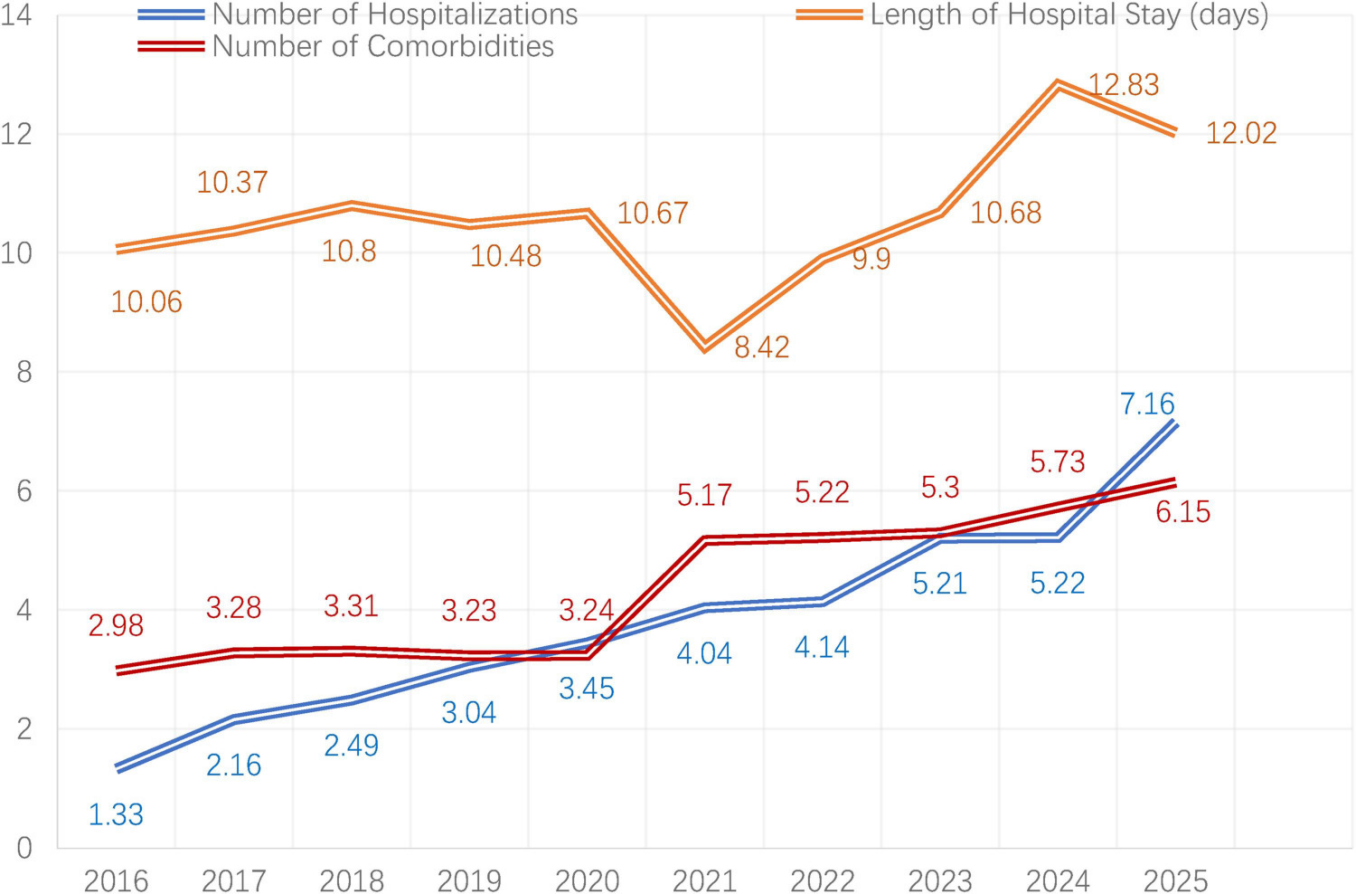
Annual trends in hospitalization metrics for COPD admissions.

Comorbidities were highly prevalent (97.80% of admissions), with 15.8% of the admissions having 1–2 comorbidities, 57.0% having 3–5, and 25.0% having ≥6. The mean comorbidity count rose from a stable approximately 3 (2016–2020) to 6.15 in 2025, with a marked increase starting in 2021 (Figure 1).

### 3.2 Hospitalization cost composition and trends

**Figure 2:**
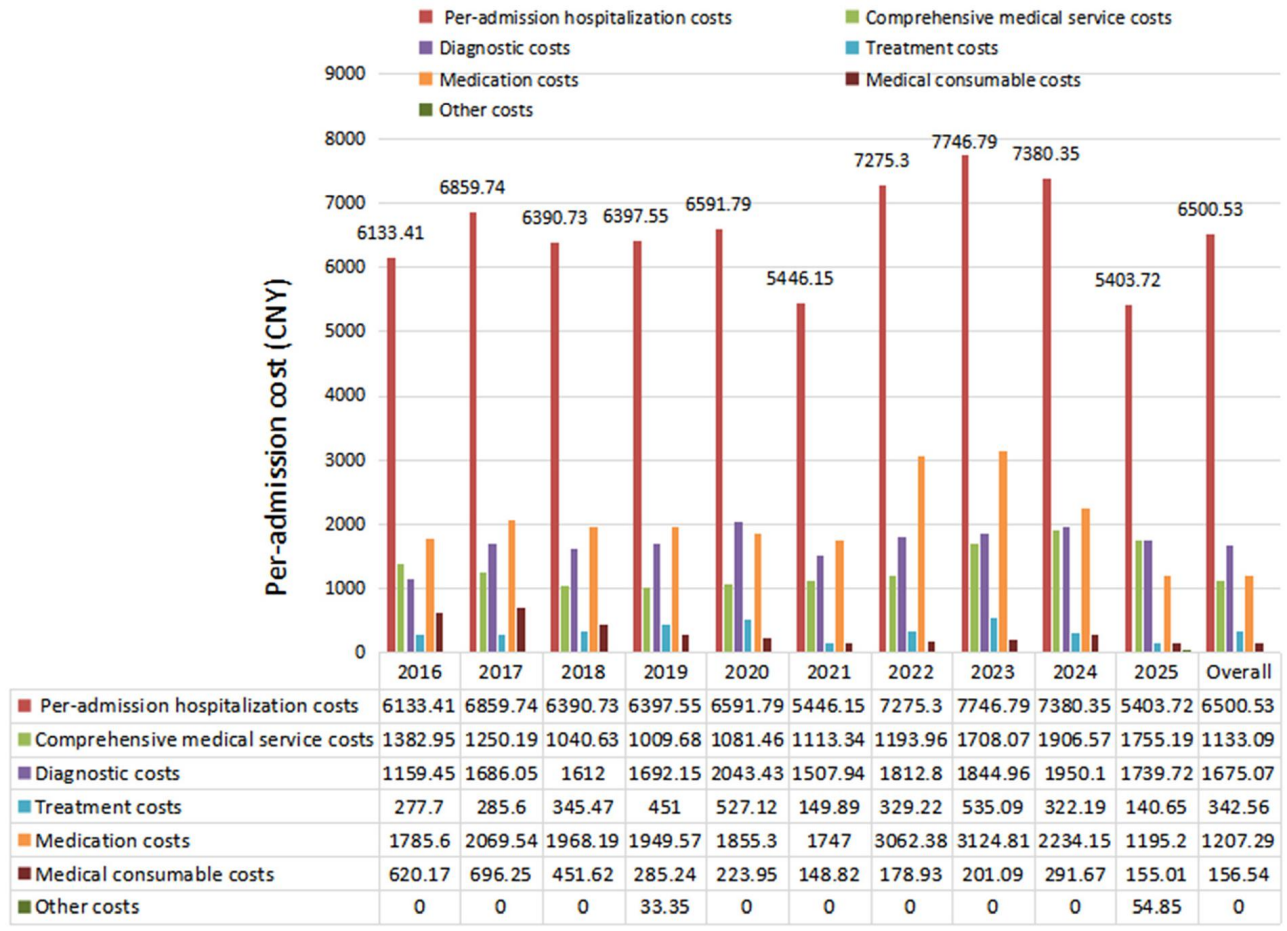
Annual trends in median per-admission cost composition for COPD admissions.

Per-admission hospitalization costs were skewed, so median values are reported to better reflect central tendency. The median total cost fluctuated between ¥5,403.72 (2025) and ¥7,746.79 (2023), with an overall 10-year median of ¥6,500.53 (range: ¥4,522.86–9,956.90), remained generally stable overall (Figure 2).

Core cost components included drug costs, comprehensive medical service costs, and diagnostic costs. Comprehensive medical service costs (median: ¥1,009.68–1,906.57) and diagnostic costs (median: ¥1,159.45–2,043.43) remained stable. Drug costs peaked at ¥3,124.81 in 2023, then declined, showing a “rise-then-fall” pattern. Consumable costs, treatment fees, and other costs contributed less and exhibited smaller fluctuations.

### 3.3 Univariate analysis of per-admission hospitalization costs for COPD admissions

The median per-admission hospitalization cost for COPD admissions was set as the dependent variable, with each indicator of baseline characteristics as independent variables for univariate analysis. Results showed statistically significant differences in the median cost among admissions with different ages, admission year, LOS, admission routes, admission statuses, discharge modes, comorbidity counts, antibiotic use, surgery/invasive procedure histories, and those complicated with hypertension, heart diseases, cerebral stroke, asthma, chronic lung diseases or kidney diseases (*P* < 0.05). Detailed data are presented in Table 1.

**Table 1:** Univariate analysis of per-admission hospitalization costs in Chinese RMB (¥).

Variables	*N* (%)	Median (P25, P75)	U/H value	*P* value
Gender			2.07 × 10^6^	0.893
Male	3,617 (75.9)	6,519.90 (4,505.77, 10,014.50)		
Female	1,149 (24.1)	6,448.25 (4,597.72, 9,760.91)		
Age (years)			19.55	<0.001
<60	337 (7.1)	5,814.38 (4,135.78, 8,442.16)		
60–69	1,161 (24.4)	6,403.86 (4,479.71, 9,567.36)		
70–79	1,662 (34.9)	6,514.74 (4,502.25, 10,072.44)		
≥80	1,606 (33.7)	6,653.40 (4,697.79, 10,525.68)		
Marital status			3.31 × 10^5^	0.115
Married	4,611 (96.7)	6,471.89 (4,522.14, 9,933.27)		
Other	155 (3.3)	7,016.92 (4,655.88, 11,012.36)		
Admission route			2,516.54	<0.001
Outpatient	2,459 (51.6)	6,072.87 (4,315.51, 8,829.32)		
Emergency	2,307 (48.4)	7,113.26 (4,819.78, 11,311.66)		
Admission condition			201.94	<0.001
General	743 (15.6)	5,634.9 (3,993.33, 8,149.56)		
Acute	2,948 (61.9)	6,318.55 (4,458.51, 9,406.96)		
Critical	1,075 (22.6)	8,112.42 (5,450.82, 14,017.94)		
Number of hospitalizations (times)			0.15	0.927
1	1,817 (38.1)	6,485.44 (4,508.1, 9,982.62)		
2–5	2,002 (42.0)	6,560.91 (4,578.57, 9,899.32)		
≥5	947 (19.9)	6,441.6 (4,483.11, 9,921.91)		
Discharge mode			32.74	<0.001
Medical advice	4,102 (86.1)	6,437.3 (4,581.75, 9,688.53)		
Against medical advice	552 (11.6)	6,895.12 (3,980.52, 11,169.99)		
Death	66 (1.4)	13,165.4 (6,476.56, 28,892.18)		
Other	46 (1.0)	6,134.29 (4,597.21, 11,970.82)		
LOS (days)			2,516.54	<0.001
≤7	1,914 (40.2)	4,452.73 (3,364.68, 5,762.27)		
8–14	2,031 (42.6)	7,401.62 (5,725.68, 9,630.71)		
≥15	821 (17.2)	15,689.06 (11,459.45, 27,490.92)		
Comorbidity count			416.77	<0.001
≤2	860 (18)	4,998.04 (3,733.17, 6,595.36)		
3–5	2,715 (57)	6,528.24 (4,542.75, 9,914.95)		
≥6	1,191 (25)	8,161.31 (5,742.4, 12,853.9)		
Antibiotic use			2.88 × 10^5^	<0.001
No	249 (5.2)	3,872.58 (2,545.06, 5,726.82)		
Yes	4,517 (94.8)	6,637.14 (4,688.76, 10,153.81)		
History of surgery/invasive procedures			3.49 × 10^5^	<0.001
No	4,357 (91.4)	6,246.45 (4,405.65, 9,192.70)		
Yes	409 (8.6)	13,791.35 (8,378.18, 29,490.65)		
Hypertension			2.24 × 10^6^	0.004
No	3,354 (70.4)	6,376.64 (4,461.99, 9,900.49)		
Yes	1,412 (29.6)	6,760.61 (4,751.91, 10,134.24)		
Asthma			5.63 × 10^5^	<0.001
No	4,556 (95.6)	6,561.87 (4,573.12, 10,049.26)		
Yes	210 (4.4)	5,377.78 (3,819.99, 8,097.19)		
Chronic Lung Disease			2.23 × 10^6^	<0.001
No	3,014 (63.2)	6,131.52 (4,278.09, 9,243.92)		
Yes	1,752 (36.8)	7,207.38 (5,073.51, 11,333.98)		
Heart Disease			8.57 × 10^5^	<0.001
No	4,258 (89.3)	6,373.31 (4,456.32, 9,658.28)		
Yes	508 (10.7)	7,863.1 (5,464.15, 12,810.12)		
Cerebral Stroke			4.52 × 10^5^	<0.001
No	4,530 (95)	6,444.31 (4,493.23, 9,864.39)		
Yes	236 (5)	7,694.27 (5,196.39, 11,854.17)		
Kidney Disease			7.21 × 10^5^	0.004
No	4,406 (92.4)	6,457.91 (4,504.35, 9,886.18)		
Yes	360 (7.6)	7,189.47 (4,991.82, 10,785.31)		
Diabetes Mellitus			8.03 × 10^5^	0.384
No	4,390 (92.1)	6,468.29 (4,504.35, 9,992.16)		
Yes	376 (7.9)	6,695.65 (4,784.67, 9,476.51)		
Cancer			3.48 × 10^5^	0.126
No	4,626 (97.1)	6,520.08 (4,555.22, 9,972.03)		
Yes	140 (2.9)	6,278.59 (3,895.87, 9,328.61)		
Digestive System Disease			2.12 × 10^5^	0.57
No	3,564 (74.8)	6,434.28 (4,540.77, 9,978.66)		
Yes	1,202 (25.2)	6,745.65 (4,485.29, 9,885.68)		
Admission year			204.34	<0.001
2016	520 (10.9)	6,133.41 (4,205.51, 9,285.08)		
2017	296 (6.2)	6,889.74 (4,987.79, 10,358.54)		
2018	378 (7.9)	6,390.73 (4,210.67, 9,684.27)		
2019	784 (16.4)	6,397.55 (4,451.25, 9,792.01)		
2020	273 (5.7)	6,591.79 (4,828.96, 10,580.41)		
2021	431 (9)	5,446.15 (4,117.69, 6,890.76)		
2022	480 (10.1)	7,275.3 (5,576.49, 10,279.28)		
2023	646 (13.6)	7,746.79 (5,440.48, 11,491.6)		
2024	301 (6.3)	7,380.35 (5,237, 12,457.87)		
2025	657 (13.8)	5,403.72 (3,839.74, 9,396.35)		

U/H values with magnitude ≥ 100,000 are presented in scientific notation to optimize table readability.

### 3.4 Multiple linear regression analysis of per-admission hospitalization costs for COPD admissions

Given the skewed distribution of raw per-admission costs, ln transformation was applied to improve normality. Post-transformation, the skewness was 0.473 (|skewness| < 3) and kurtosis was 5.92 (|kurtosis| < 10), satisfying the “approximate normality” requirement for linear regression.

To capture both linear associations and potential non-linear effects of continuous variables, we built a multiple linear regression model with log-transformed total per-admission cost as the outcome. Independent variables included those significant in univariate analysis: age, admission year, LOS, number of comorbidities, admission route, admission status, discharge mode, antibiotic use, surgery/invasive procedure histories, and those complicated with hypertension, heart diseases, cerebral stroke, asthma, chronic lung diseases or kidney diseases. Continuous variables (age, LOS, comorbidity count and number of hospitalizations) were included as continuous terms in the model.

The overall model was statistically significant (*F* = 314.3, *P* < 0.001). Residuals approximated a normal distribution, and variance inflation factors (VIFs) for all independent variables were <10, confirming no multicollinearity. These results indicated the data satisfied the assumptions of multiple linear regression, and the model exhibited good fit and high reliability.

Significant independent variables in the final model included admission year (2017, 2021–2025), age, LOS, comorbidity count, emergency admission, acute/critical admission status, discharge mode (death), history of surgery/invasive procedures, antibiotic use, and those complicated with heart diseases and cerebral stroke. All of these variables were positively associated with ln-transformed total per-admission cost, with results shown in Table 2 (only variables with *P* < 0.05 are shown). The adjusted *R*^2^ was 0.703, indicating that the model explained 70.3% of the variance in log-transformed costs. As a sensitivity analysis addressing potential endogeneity concerns related to LOS, we fitted a model excluding LOS. The direction and statistical significance of all core predictors remained consistent with the main model, confirming the robustness of our findings.

**Table 2:** Multiple linear regression of ln-transformed per-admission hospitalization costs for COPD admissions.

Variables	β	EXP(β) (GMR)	Lower 95% CI	Upper 95% CI	*P Value*
Intercept	7.215	1,360.231	1,116.429	1,657.272	<0.001
Age (years)	0.004	1.004	1.001	1.008	0.008
LOS (days)	0.038	1.038	1.036	1.041	<0.001
Admission route (ref = outpatient)					
Emergency	0.079	1.082	1.057	1.108	<0.001
Admission condition(ref = general)					
Acute	0.047	1.048	1.012	1.086	0.009
Critical	0.183	1.201	1.150	1.255	<0.001
Discharge mode(ref = discharged on medical advice)					
Death	0.323	1.381	1.250	1.525	<0.001
Comorbidity count	0.040	1.041	1.028	1.054	<0.001
History of surgery/invasive procedures (ref = No)	0.449	1.567	1.500	1.637	<0.001
Antibiotic use (ref = No)	0.417	1.517	1.439	1.599	<0.001
Heart Disease (ref = No)	0.051	1.052	1.011	1.094	0.011
Cerebral Stroke (ref = No)	0.092	1.096	1.039	1.156	<0.001
Admission year (ref = 2016)					
2017	0.123	1.131	1.069	1.198	<0.001
2021	−0.089	0.914	0.866	0.965	0.001
2022	0.161	1.174	1.114	1.238	<0.001
2023	0.219	1.245	1.184	1.310	<0.001
2024	0.076	1.079	1.015	1.147	0.015
2025	−0.116	0.890	0.844	0.940	<0.001

EXP(β) = exponentiated natural logarithmic coefficient (GMR). Table 2 presents only significant variables (*P* < 0.05) to optimize readability; full results are available upon request.

Regression coefficients, 95% confidence intervals (95% CI), and statistical significance are detailed in Table 2.

### 3.5 Quantile regression analysis of per-admission hospitalization costs for COPD admissions

To examine the heterogeneous effects of influencing factors across different expenditure tiers, we performed quantile regression on unadjusted per-admission total costs, using variables that were statistically significant in the univariate analysis as predictors.

The quantile regression model was statistically significant at the 10th, 50th, and 90th percentiles (all intercept *P* < 0.001), confirming its ability to detect heterogeneous effects of predictors across cost tiers. Admission year effects were heterogeneous across quantiles: the cost-increasing effect of 2022–2024 and cost-decreasing effect of 2021/2025 were most pronounced in the high-cost (90th percentile) subgroup, indicating that temporal factors had a stronger impact on expensive admissions. LOS, comorbidity count, history of surgery/invasive procedures, discharge mode (death), emergency admission, and critical admission status were positively associated with per-admission total costs across all quantiles (all *P* < 0.01), with regression coefficients increasing across higher quantiles. This indicates that these factors have a disproportionately stronger association with per-admission costs among high-cost admissions. Notably, the coefficient for “Other” discharge mode at the 90th percentile was notably large with a relatively high standard error, likely reflecting estimation instability due to the small sample size of this category.

Furthermore, age, antibiotic administration, and comorbidity with cerebral stroke were significantly and positively associated with hospitalization costs only in the low and middle cost subgroups (10th and 50th percentiles), with no significant independent effect observed in the highest-cost (90th percentile) subgroup. In contrast, comorbidity with hypertension and chronic lung diseases showed a significant positive association only in the middle cost subgroup (50th percentile). These observations highlight the heterogeneity in factors associated with per-admission costs across different expenditure tiers (Table 3).

**Table 3:** Quantile regression analysis of untransformed total hospitalization costs for COPD admissions.

Variables	10th percentile	*P value*	50th percentile	*P value*	90th percentile	*P value*
Intercept	−2,842.21 (466.76)	<0.001	−3,844.18 (478.98)	<0.001	−4,202.3 (930.71)	<0.001
Age (years)	25.98 (8.2)	0.002	27.59 (7.52)	<0.001	21.12 (15.03)	0.16
LOS (days)	444.56 (21.36)	<0.001	751.74 (28.27)	<0.001	1,325.57 (52.35)	<0.001
Comorbidity count	140.36 (31.96)	<0.001	192.51 (30.01)	<0.001	496.82 (80.3)	<0.001
Admission route (ref = outpatient)						
Emergency	349.33 (55.93)	<0.001	406.34 (54.38)	<0.001	740.4 (112.44)	<0.001
Admission condition (ref = general)						
Acute	173.92 (65.83)	0.008	341.78 (67.25)	<0.001	50.31 (152.68)	0.742
Critical	770.91 (102.84)	<0.001	1,267.9 (100.18)	<0.001	2,074.89 (237.17)	<0.001
Discharge mode (ref = discharged on medical advice)						
Death	340.52 (131.64)	0.01	971.38 (255.39)	<0.001	1,495.22 (473.7)	0.002
Other	425.63 (1,158.91)	0.713	2,556.42 (1,538.79)	0.097	30,897.68 (12,091)	0.011
Against medical advice	−43.97 (118.7)	0.711	584.91 (120.6)	<0.001	1,562.9 (455.96)	<0.001
History of surgery/invasive procedures (ref = No)	1,831.26 (294.83)	<0.001	3,278.75 (329.17)	<0.001	23,263.69 (1,956.99)	<0.001
Antibiotic use (ref = No)	1,121.42 (77.94)	<0.001	925.25 (166.1)	<0.001	222.74 (217.17)	0.305
Hypertension (ref = No)	22.6 (55.52)	0.684	156.43 (54.54)	0.004	−233.07 (154.37)	0.131
Heart diseases (ref = No)	47.83 (130.05)	0.713	140.48 (114.61)	0.22	156.31 (177.45)	0.378
Cerebral stroke (ref = No)	335.68 (118.33)	0.005	578.18 (147.08)	<0.001	523.52 (392.08)	0.182
Asthma (ref = No)	−63.57 (90.67)	0.483	−165.88 (98.86)	0.093	−243.83 (152.82)	0.111
Chronic lung diseases (ref = No)	29.9 (64.82)	0.645	217.9 (65.45)	<0.001	264.84 (142.49)	0.063
Kidney disease (ref = No)	20.02 (133.89)	0.881	−102.21 (82.83)	0.217	−136.49 (271.04)	0.615
Admission year (ref = 2016)						
2017	194.12 (131.78)	0.141	806.22 (106.75)	<0.001	805.42 (252.8)	0.001
2018	144.51 (107.51)	0.179	38.71 (123.5)	0.754	406.65 (231.55)	0.079
2019	89.17 (114.95)	0.438	−188.03 (92.73)	0.043	−111.2 (148.53)	0.454
2020	270.34 (123.5)	0.029	196.14 (114.2)	0.086	−48.61 (224.53)	0.829
2021	−76.7 (91.83)	0.404	−228.4 (96.02)	0.017	−593.77 (185.39)	0.001
2022	1,045.22 (139.66)	<0.001	1,012.3 (87.36)	<0.001	1,029.88 (278)	<0.001
2023	1,266.36 (107.15)	<0.001	1,550.96 (100.09)	<0.001	1,541.08 (211.03)	<0.001
2024	468.21 (132.31)	<0.001	599.57 (133.24)	<0.001	1,579.94 (617.42)	0.011
2025	−357.96 (111.42)	0.001	−438.57 (109.63)	<0.001	−743.57 (211.49)	<0.001

Standard errors in parentheses.

## 4 Discussion

GOLD 2026 underscores the substantial global disease burden of COPD, reiterating its status as the third leading cause of mortality worldwide. It further highlights that COPD prevalence, mortality, and associated socioeconomic burden are not only rising steadily but also marked by striking geographic heterogeneity (13–15). Projections suggest that by 2050, direct and indirect economic costs associated with COPD will reach $24.35 trillion and $15.43 trillion, respectively, with medical spending driven by acute exacerbations set to exhibit the most pronounced growth (16).

Fang et al. analyzed COPD patient data from a tertiary hospital in Guizhou spanning 2012–2021, reporting a sustained upward trend in hospitalization costs over the decade (17). In contrast, Xue et al. observed a marked reduction in total COPD hospitalization costs between 2018 and 2022, with a 1.82% monthly average decline following the rollout of DRG-based payments (18).

In this study, we analyzed data from 4,766 eligible COPD-related hospitalization events at a tertiary general hospital in Guangxi, China, spanning the 10-year period from 2016 to 2025. Our primary aim was to systematically examine the long-term dynamic trends in average per-admission costs and the heterogeneous impacts of associated factors. Notably, our findings contrast with those of prior studies: while average per-admission COPD hospitalization costs showed minor annual fluctuations over the study decade, they remained broadly stable overall. We tentatively hypothesize this stability to the potential regulatory effects of national health insurance policies in China. For instance, the nationwide rollout of centralized volume-based procurement for pharmaceuticals and medical supplies may have substantially reduced the prices of core COPD therapies, thereby curbing the rapid escalation of drug expenditures (19). Concurrently, Diagnosis Related Groups/Diagnosis-Intervention Packet (DRG/DIP) payment reforms may have incentivized healthcare facilities to proactively standardize clinical practices, potentially mitigating cost inflation associated with overutilization of medical services from the outset (20).

Notably, admission year effects were not universal: only specific years (2017, 2021–2025) showed significant differences in costs compared with 2016 after adjustment, which align closely with the timelines and impacts of key healthcare policies and public health events. In 2017, the overall improvement in local diagnosis and treatment capacity, together with the gradual adjustment of medical service prices, contributed to a moderate increase in hospitalization costs. In 2021, strict COVID-19 pandemic control measures shortened hospital stays and reduced non-essential admissions, leading to a notable decline in costs. From 2022 to 2024, the relaxation of pandemic restrictions and full recovery of routine medical services drove a rebound in standardized care and corresponding cost growth. In 2025, DRG/DIP payment was fully implemented and refined across all county-level and primary care facilities in Guangxi, strengthened precision cost control effectively curbed excessive medical expenses and resulted in a second reduction in hospitalization costs.

Consistent with this policy-driven cost moderation, international comparisons reveal that China's median COPD hospitalization costs are lower than those in Western countries but higher than in several Asian developing nations (21–23). This cross-country disparity may in part reflect the inherent regional heterogeneity of healthcare costs within China, while also underscoring the potential role of national health insurance policies—including the procurement and payment reforms referenced above—in curbing excessive expenditure growth and shaping the current cost landscape.

Our cost composition analysis reveals that drug costs, comprehensive medical service costs, and diagnostic costs collectively account for over 70% of total hospitalization costs, with drug expenditures remaining disproportionately high. This pattern aligns closely with the pathophysiological nature of COPD, a chronic inflammatory disorder requiring long-term pharmacotherapy (1). However, it also highlights gaps in clinical pathway optimization and the need for more precise medication selection. Notably, we could not identify the specific drugs driving the 2023 cost peak due to unstratified total drug cost data, a key limitation of this analysis. There is an urgent need to optimize the cost structure through the development of standardized clinical protocols.

Among the factors we analyzed, age is a critical factor associated with both COPD onset and higher hospitalization costs, with its underlying mechanisms meriting further investigation. In our cohort, 92.9% of admissions were for patients aged ≥60 years — consistent with the global epidemiological profile of COPD, a disease that disproportionately affects older adults (24). Elderly patients typically exhibit age-related physiological decline and impaired immune function, alongside a higher prevalence of comorbidities such as hypertension, diabetes, and coronary heart disease. In our study, comorbidity prevalence reached 97.80% of admissions, with the average comorbidity count rising annually to 6.15 by 2025. This finding directly reflects the susceptibility of the elderly population to multi-organ functional degeneration and concurrent illnesses (25).

Increasing age not only significantly elevates the risk of COPD onset but also leads to increased disease severity. Elderly patients with acute exacerbations are more prone to complications such as respiratory failure and septic shock. These complications require more intensive examinations, higher-level life support (e.g., non-invasive ventilation, intensive care), and multitargeted therapies, which in turn drive up healthcare costs (26). This finding highlights the need to prioritize elderly high-risk groups in COPD prevention and control. We suggest implementing preventive measures such as early screening, smoking cessation interventions, and pulmonary rehabilitation to delay disease progression and reduce hospitalization demand and cost burden at the source.

The comorbidity count constitute another is another important factor associated with higher hospitalization costs. Our regression analyses demonstrate a significant positive association between comorbidity burden and hospitalization costs, with a more pronounced association observed in the high-cost subgroup (90th percentile). COPD patients often have concurrent cardiovascular diseases, metabolic disorders, and psychiatric conditions. These comorbidities not only increase the complexity of clinical management but also raise the risk of drug-drug interactions and cross-deterioration of conditions, leading to redundant examinations and prolonged treatment courses (27, 28). For example, COPD patients complicated by heart failure require simultaneous monitoring of cardiac and pulmonary function and adjustment of the balance between diuretics and bronchodilators, which significantly increases diagnostic and treatment complexity and costs. This underscores the importance of early screening and management of comorbid patients, as well as the establishment of a multidisciplinary team (MDT) collaboration mechanism involving respiratory, cardiology, and endocrinology departments (29, 30). Through unified assessment and individualized treatment planning, we can reduce ineffective healthcare expenditures and improve patient outcomes.

Furthermore, our analyses confirm that LOS, prior surgery/procedures, and admission for acute critical illness are significant factors associated with higher hospitalization costs. As a robust predictor of hospitalization costs, prolonged LOS is directly linked to disease complexity and delayed recovery. It necessitates ongoing utilization of medical resources (e.g., oxygen therapy, nursing care) and elevates the risk of nosocomial infections, thereby perpetuating a cost-LOS vicious cycle. Standardizing clinical pathways and reducing unnecessary LOS are critical to breaking this cycle. We believe that by implementing the Chinese Expert Consensus on the Diagnosis and Treatment of Acute Exacerbations of Chronic Obstructive Pulmonary Disease and optimizing the full pathway of “admission assessment, precision treatment, early rehabilitation, and post-discharge follow-up”, unnecessary hospitalization duration can be effectively reduced (31, 32).

Notably, we observed a distinct fluctuation in average LOS during the study period, particularly a significant reduction to 8.42 days in 2021, which coincided with the strict implementation phase of China's dynamic zero-COVID policy. A possible explanation for this LOS reduction is the pandemic-related policy measures, and as LOS is an important factor associated with hospitalization costs for COPD patients, this pandemic-related policy shift represents a major healthcare system-level confounder that may have potentially influenced the observed trends in costs and associated factors. This temporal context should be carefully considered when interpreting the study findings.

Prior surgery/procedures (e.g., bronchoscopic interventions, thoracentesis) and admission for acute critical illness are directly linked to costs of high-value consumables and advanced life support. These patients are mostly critically ill, and we recommend priority optimization of clinical decision-making through the MDT mechanism to avoid overmedicalization.

Additionally, this study found that antibiotic use was positively correlated with hospitalization costs, with a heterogeneous effect across cost tiers: the association was significant in low- and middle-cost patients but non-significant in the highest-cost subgroup, likely due to near-universal antibiotic use in severe, high-cost patients that eliminates its discriminative power for cost variation. This emphasizes the need to strictly adhere to etiological diagnosis principles and ensure precision use of anti-infective agents, particularly in mild-to-moderate patients, to reduce costs and the emergence of drug-resistant bacteria.

From a regional epidemiological standpoint, Guangxi is among the Chinese provinces with the highest COPD prevalence. Surveillance data from 2014 to 2024 indicate that the annual average COPD mortality rate in Guangxi is 54.19 per 100,000 population, accounting for 8.12% of all-cause deaths and reflecting a substantial local disease burden. The Healthy Guangxi Initiative: Action Plan for the Prevention and Control of Chronic Respiratory Diseases (2024–2030) also identifies COPD as a key disease for prevention and control, further confirming its high prevalence through policy positioning (33, 34).

The patients included in this study are predominantly elderly, with relatively limited healthcare payment capacity. The cost pressure from high comorbidity rates, prolonged LOS, and excessive proportion of drug costs is likely to exacerbate the economic burden on patients’ families. Building on these findings, we propose the following targeted strategies:

① Prioritize elderly high-risk populations by establishing a hospital-community collaborative framework for early prevention and long-term disease management, implementing validated COPD screening tools, and enhancing patient education on disease awareness and self-management to mitigate acute exacerbation risk.
② Optimize clinical pathways, standardize the diagnosis and treatment process for acute exacerbations of COPD, and prioritize the use of drugs procured through national centralized volume-based procurement to improve the cost structure characterized by an excessive proportion of drug costs.
③ Strengthen the MDT collaboration mechanism, enhance the comprehensive management of patients with comorbidities, shorten average LOS, and improve diagnostic and treatment efficiency and cost-effectiveness.
④ Leverage the fully implemented DRG payment system, dynamically adjust grouping and payment standards, and guide healthcare facilities to proactively standardize clinical practices and reasonably control costs.

### 4.1 Limitations of the study

This study used quantile regression to explore heterogeneous cost drivers for COPD hospitalizations, but several key limitations need to be noted. First, we lacked direct measures of COPD severity (e.g., spirometry, GOLD stage), so variables like LOS, comorbidity count, and emergency admission only served as proxies for severity, not independent cost predictors. Second, our analysis was explicitly admission-based (per admission event), not patient-level. All counts of prior hospitalizations are defined per admission, not per unique patient, eliminating ambiguity in the unit of analysis. Third, as a single-center study, our findings cannot be generalized to other regions or hospital levels. Fourth, we could not break down drug costs by specific type, and lacked insurance reimbursement data to assess patient out-of-pocket expenses. Fifth, as a retrospective study using routine clinical records, our analysis is subject to information bias, incomplete data, and unmeasured confounding, and cannot infer causality. Future multicenter studies with complete severity data and stratified cost categories will help validate these results.

## 5 Conclusion

In conclusion, per-admission COPD hospitalization costs remained stable over the decade, with drug, medical service, and diagnostic costs as the primary components. Factors such as age, comorbidity count, LOS, invasive procedures, and admission for acute critical illness were significantly associated with higher per-admission costs, particularly among high-cost admissions. This study lays a foundation for the targeted cost containment and optimization of clinical management strategies for COPD-related hospitalizations.

## Data Availability

The raw data supporting the conclusions of this article will be made available by the authors, without undue reservation.
